# Enhanced T Cell Lymphoma in NOD.Stat5b Transgenic Mice Is Caused by Hyperactivation of Stat5b in CD8^+^ Thymocytes

**DOI:** 10.1371/journal.pone.0056600

**Published:** 2013-02-14

**Authors:** Bo Chen, Bing Yi, Rui Mao, Haitao Liu, Jinhua Wang, Ashok Sharma, Stephen Peiper, Warren J. Leonard, Jin-Xiong She

**Affiliations:** 1 Center for Biotechnology and Genomic Medicine, Medical College of Georgia, Georgia Regents University, Augusta, Georgia, United States of America; 2 Sino-American Institute for Translational Medicine, Nanjing University of Technology, Nanjing, People's Republic of China; 3 Jiangsu Cancer Hospital, Nanjing, People's Republic of China; 4 Department of Pathology, Jefferson University, Philadelphia, Pennsylvania, United States of America; 5 National Heart, Lung, and Blood Institute, National Institutes of Health, Bethesda, Maryland, United States of America; Emory University, United States of America

## Abstract

Activation of signal transducers and activators of transcription (STAT) proteins may be critical to their oncogenic functions as demonstrated by the development of B-cell lymphoma/leukemia in transgenic (TG) mice overexpressing a constitutively activated form of Stat5b. However, low incidence of CD8^+^ T cell lymphoma was observed in B6 transgenic mice overexpressing a wild-type Stat5b (B6.Stat5b^Tg^) despite of undetectable Stat5b phosphorylation and the rate of lymphomagenesis was markedly enhanced by immunization or the introduction of TCR transgenes [1]. Here, we report that the wild-type Stat5b transgene leads to the acceleration and high incidence (74%) of CD8^+^ T cell lymphoblastic lymphomas in the non-obese-diabetic (NOD) background. In contrast to the B6.Stat5b^Tg^ mice, Stat5b in transgenic NOD (NOD.Stat5b^Tg^) mice is selectively and progressively phosphorylated in CD8^+^ thymocytes. Stat5 phosphorylation also leads to up-regulation of many genes putatively relevant to tumorigenesis. Treatment of NOD.Stat5b^Tg^ mice with cancer chemopreventive agents Apigenin and Xanthohumol efficiently blocked lymphomagenesis through reduction of Stat5 phosphorylation and genes up-regulated in the NOD.Stat5b^Tg^ mice. These results suggest that NOD genetic background is critical to the Stat5b-mediated lymphomagenesis through regulation of Stat5 hyperactivation. NOD.Stat5b^Tg^ mouse is an excellent model for studying the molecular mechanisms underlying lymphomagenesis and testing novel chemoprevention strategies.

## Introduction

The mammalian STAT family contains seven members that play diverse roles in embryonic cell development, differentiation, proliferation, migration, survival and apoptosis. Their activities are regulated by different cytokines, hormones and growth factors. In lymphoid cells, STAT1, 3, 5a and 5b are believed to be responsible for cell survival and growth, while STAT2, 4 and 6 are preferentially involved in differentiation [2]. STATs exist as monomers in the cytoplasm before receptor activation [3]. Cytokine stimulation leads to Janus kinase (JAK) activation followed by phosphorylation of tyrosine residues in the cytoplasmic domain of the cytokine receptor. Activated JAKs, which phosphorylate the C-terminal tyrosine residue of STATs, lead to STAT dimer formation by the intermolecular interactions of the SH2 domain and the phosphorylated tyrosine [4]. Once dimerized, STATs dissociate from the receptors and translocate to the nucleus where they bind to target DNA and regulate gene expression.

STAT5B, a member of the STAT family, has been implicated as an oncogene, consistent with its role in cell proliferation and survival [1], [5], [6]. It can be activated by multiple cytokines including IL-2, IL-3, IL-5, IL-7, IL-9 and IL-15, various growth factors [7] as well as IL-21, IL-31, TSLP, IL-4. IL-7 induced Stat5 can synergize with the Flt-3 signaling pathway [8]. Moreover, Stat5 plays a suppressive role on Th17 differentiation [9]. STAT5 can be activated through phosphorylation by tyrosine kinases. The roles of STAT5 in tumorigenesis and drug resistance have been increasingly recognized in the last few years.

Over-expression of STAT5 and phosphorylated STAT5 have been reported for a variety of cancers including breast, prostate, lung, head and neck, liver, melanoma and lymphomas [10], [11], [12], [13]. Constitutive activation of STAT5 also predicts drug resistance in T cell lymphoma [14]. However, STAT5 activity is associated with better prognosis for survival in breast cancer [15], [16]. Clearly, the role of STAT5 in cancer is still poorly understood and requires further investigation.

The roles of STAT5 in tumorigenesis have also been demonstrated in animal studies. C57BL/6 (B6) mice with a wild-type Stat5b transgene, which is conditionally over-expressed in T, B and NK cells, develops low incidence of CD8^+^ lymphoblastic lymphoma with characteristics of T cell acute lymphoblastic leukemia/lymphoma (T-ALL) [1]. However, the molecular mechanism underlying the disease remains elusive because Stat5b phosphorylation was surprisingly not observed in these transgenic mice with or without lymphomas.

T-ALL is a neoplastic disorder of the lymphoblast committed to the T-cell lineage [17], [18]. There are racial differences for the incidence, characteristics and outcome of T-ALL, suggesting a role of genetic background in lymphomagenesis [19]. T-ALL has been linked to JAK-STAT pathway [20].

Higher baseline phosphorylation of Stat5b was recently reported for NOD mice [21], [22]. We have recently reported reduced incidence of type 1 diabetes in the NOD Stat5b transgenic mice associated with increased levels of CD4^+^ regulatory T cells [23], in which Stat5 plays a critical role. Therefore, we hypothesized that transgenic Stat5b in the NOD genetic background may enhance lymphoma development. Indeed, NOD mice with the Stat5b transgene (NOD.Stat5b^Tg^) developed high incidence of CD8^+^ T cell lymphoma with earlier age of onset. We further demonstrate that activation of Stat5b in CD8^+^ T cells in the NOD genetic background is responsible for lymphomagenesis and prevention of lymphomagenesis using two chemoprevention agents correlates with the reduction of Stat5b phosphorylation and its regulated genes.

## Results

### NOD.Stat5b^Tg^ mice develop high incidence of CD8^+^ lymphoblastic lymphoma

**Figure 1:**
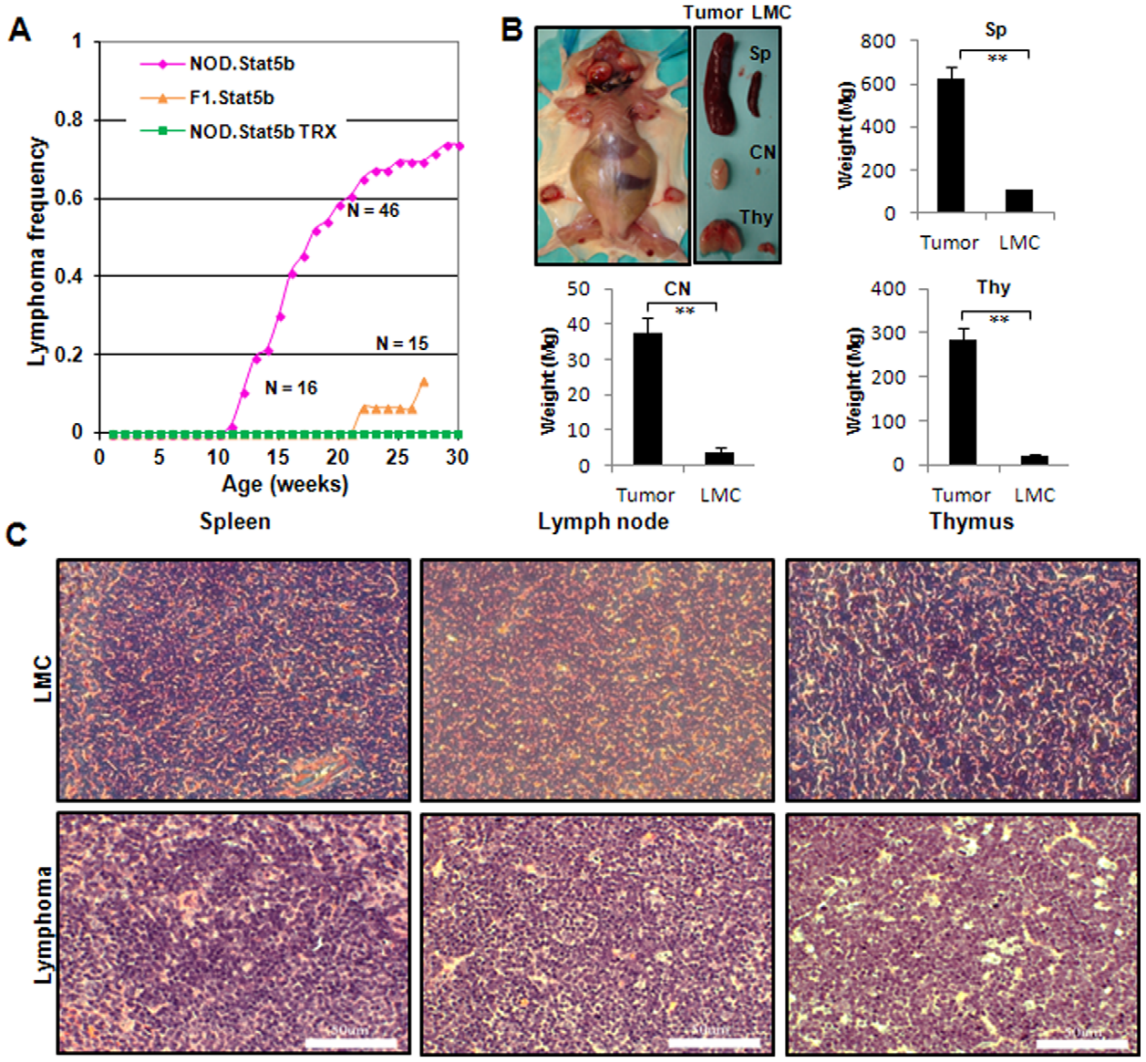
Lymphoblastic lymphoma in Stat5b transgenic mice. (A) Progression of lymphoma observed in the B6xNOD F1.Stat5b^Tg^ mice and NOD.Stat5b^Tg^ mice with or without chemoprevention treatment. The lymphoma incidence is significantly different between F1 mice and NOD.Stat5b^Tg^ mice (p<0.001). (B) Overview of a mouse that has enlarged thymus, spleen, and lymph nodes (left panel). Enlarged spleen (SP), cervical lymph node (CN) and thymus (Thy) are compared to littermate controls (right panel). **, p<0.01, compared with that of littermate controls. (C) H&E staining of spleen, cervical node and thymus from a NOD.Stat5b^Tg^ mouse with lymphoma and its littermate control (LMC). Scale bars represent 50 um.

In order to examine the effect of Stat5b transgene and genetic background on lymphomagenesis, we crossed B6.Stat5b^Tg^ mice with NOD mice and generated F1 Stat5b transgenic mice, designated as F1.Stat5b^Tg^. Approximately 13% of these F1 mice developed lymphoma by 30 weeks of age (Fig. 1a), similar to the incidence reported for Stat5b transgenic mice in the B6 background (∼12%) [1], [24]. We also generated a NOD Stat5b transgenic line (NOD.Stat5b^Tg^) through 21 generations of backcrossing with NOD mice. The NOD.Stat5b^Tg^ mice spontaneously developed lymphoma. Lymphomas were observed as early as 10 weeks of age and reached an incidence of ∼74% by 30 weeks of age (Fig. 1a). Mice with lymphomas have typically enlarged thymus, spleen, and lymph nodes, especially the cervical lymph node comparing to littermate controls (LMC) (Fig. 1b). Therefore, genes in the NOD genome can significantly increase the incidence of lymphoma and accelerate the disease when compared to the B6 or NOD/B6 F1 mice.

The lymphomas in NOD.Stat5b^Tg^ mice were composed of sheets of intermediate-sized, blastic lymphoid cells that effaced the normal architecture of lymph nodes, spleen, and the thymus. The cellular morphology and the pattern of involvement of lymphoid organs mimic the features of lymphoblastic lymphoma in humans (Fig. 1c).

**Figure 2:**
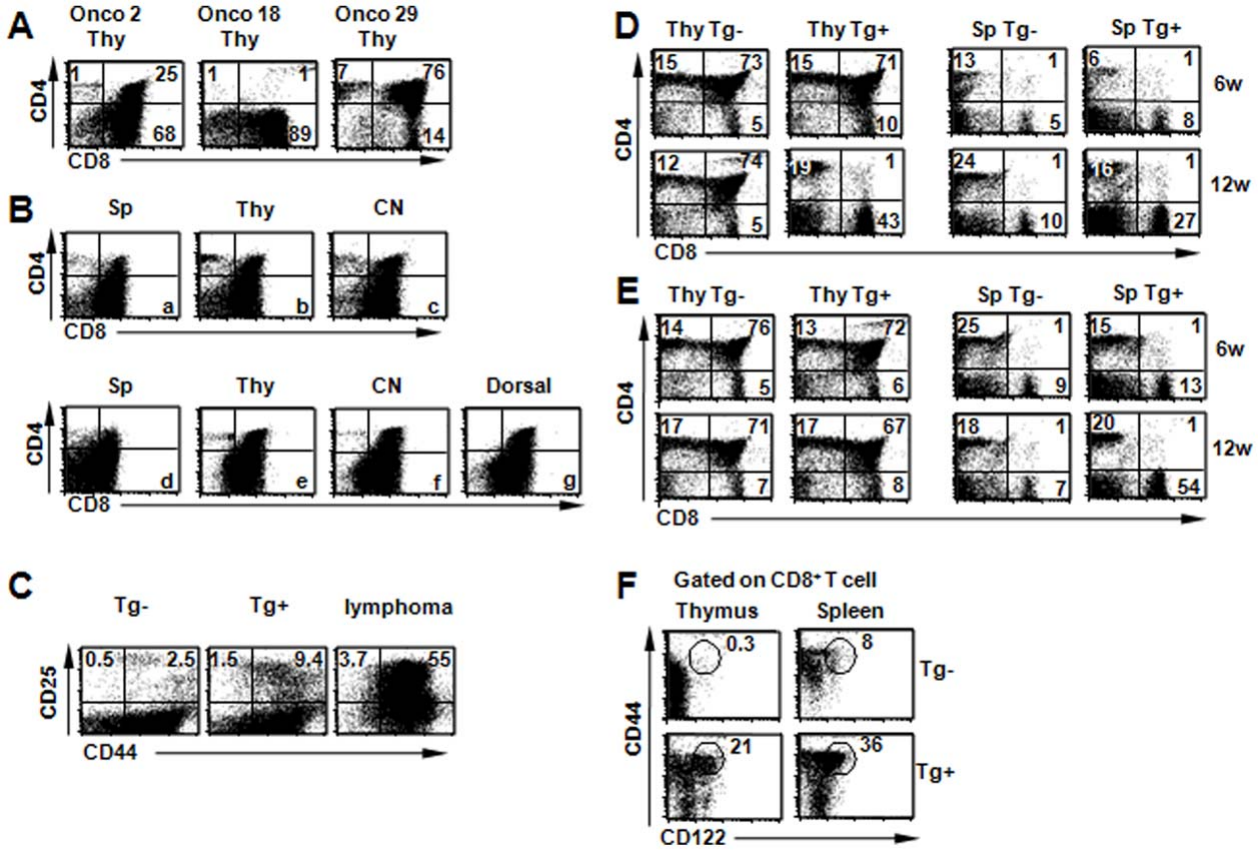
Phenotypes of CD8^+^ lymphomas and T cell development in NOD.Stat5b^Tg^ mice. (A) Three different types of lymphoma cells are illustrated. The vast majority of lymphomas (57/60) are similar to the Onco 2 mouse, e.g., with CD4^+^CD8^+^ double positive and CD8^+^ single positive thymocytes. Two mice with lymphomas had predominantly CD8^+^ single-positive thymocytes (Onco 18) and one mouse had predominantly CD4^+^CD8^+^ double-positive thymocytes (Onco 29). (B) Phenotypes of metastatic tumors. Tumor phenotypes are identical in different lymphoid organs including spleen (SP), thymus (Thy) and cervical lymph node (CN) (a, b, c). Tumor cells (5×10^6^) from thymus of the mouse as shown in b were subcutaneously injected at the back of regular NOD mice. Tumor formation was observed at the site of injection 10–20 days later and tumor cells also migrate to other lymphoid organs including spleen (d), thymus (e) and cervical node (f). Tumor phenotypes are identical at the injection site (g, dorsal) and other lymphoid organs. (C) CD25 expression in thymus of NOD.Stat5b^Tg^ mice and littermate controls at 6 weeks and lymphoma population in cervical node. (D) T cell phenotypes in the thymus (Thy) and spleen (Sp) of NOD.Stat5b^Tg^ mice and littermate controls at 6 weeks (6 w) and 12 weeks (12 w) of age. (E) T cell phenotypes in the thymus (Thy) and spleen (Sp) of NOD/B6 F1.Stat5b^Tg^ mice and littermate controls at 6 weeks (6 w) and 12 weeks (12 w) of age. (F) CD44 and CD122 expression of CD8^+^ T cells from spleens and thymi of NOD.Stat5b^Tg^ mice (16 weeks of age). Representative data are shown from 1 of 4 similar experiments.

Of the 60 mice with lymphomas analyzed by flow cytometry, 57 mice had both CD4^+^CD8^+^ double positive and CD8^+^ single positive T cells, while two mice with lymphomas had predominantly CD8^+^ cells and one mouse had predominantly CD4^+^CD8^+^ double positive cells in the thymus, spleen, and lymph nodes (Fig. 2a). Subcutaneous injection of malignant cells into regular NOD recipient mice resulted in tumor formation at the site of injection and migration to other lymphoid organs. Cells from these tumor masses and metastatic tumors had CD4/CD8 flow cytometric profiles indistinguishable from the original transplanted donor cells (Fig. 2b).

### NOD genome synergizes with Stat5b to promote CD8^+^ thymocyte expansion

Mice lacking both Stat5a and Stat5b (displaying an N-terminally truncated STAT5) have fewer pro-B and pro-T cells and are completely devoid of T cells [25], [26]. NOD.Stat5b^Tg^ mice also showed perturbations in lymphocyte development. There were many more B220^+^ B cells in the bone marrow of transgenic mice than LMC at six weeks (4.5-fold), 10 weeks (5.6-fold) and 16 weeks (2-fold) of age (Table 1). In the spleens at 6 weeks of age, there were slightly higher numbers of CD4^+^ T cells (1.2-fold) and B cells (1.4-fold) but much higher number of CD8^+^ single-positive T cells (3.9-fold) in NOD.Stat5b^Tg^ than LMC (Table 1). While the differences between transgenic mice and LMC increased slightly for CD4^+^ T cells and B cells as mice age, the difference in CD8^+^ T cells between transgenic mice and LMC increased to more than 10-fold by 16 weeks of age due to the dramatic expansion of CD8^+^ T cells in the transgenic mice (Table 1).

**Table 1:** Total cell numbers in the thymus, bone marrow and spleen of NOD.Stat5b transgenic mice and littermate controls.

		LMC			Transgenic	
Cell types	6 wk	10 wk	16 wk	6 wk	10 wk	16 wk
**Thymus**						
CD4	14.1±0.8	10.8±0.7	9.3±0.2	14±0.3#	17.8±0.7**	18.2±1.3*
CD8	4.2±0.5	3.3±0.2	3.4±0.4	9.8±0.5**	18.9±0.7**	39.7±3.7**
DP	67.7±3.6	88.7±4.5	52.5±1.8	69.4±1.3#	91±4.0#	1.5±0.3**
DN	7.8±0.5	5±0.4	5±0.6	7.4±0.6#	7.2±0.8*	28.6±2.4**
**Bone marrow**						
B220	4.1±0.4	4.7±0.6	4.8±0.4	18.2±2.1**	26.4±3.2**	9.5±1.3**
**Spleen**						
CD4	7.1±0.4	9.2±0.6	11±0.4	8.4±0.2*	12.7±0.6**	16.8±0.6**
CD4+CD25+	0.5±0.1	0.8±0.1	0.7±0.1	0.6±0.1**	1.1±0.1**	1.5±0.1**
CD4+CD25−	6.6±0.4	8.5±0.5	10.4±0.4	7.7±0.3#	11.6±0.5**	15.4±0.6**
CD8	2.5±0.3	3.7±0.2	3.8±0.6	9.7±0.6**	16.7±1.5**	38.8±2.0**
CD3	10.9±0.6	17.4±1.0	19.2±1.3	19.8±0.5**	47.3±4.5**	63.6±3.2**
B220	10.2±0.5	13.8±0.5	18±1.1	14.6±1.6#	33.1±2.6**	49.2±4.1**

Cell numbers (10^6^) are the averages for NOD.Stat5b^Tg^ transgenic mice and their non-transgenic littermate control mice (LMC). Mice at 6 weeks (6 wk), 10 weeks (10 wk) and 16 weeks (16 wk) of age were examined. Student t tests were used to assess statistical significance.

# , *P*>0.05;

* , *P*<0.05;

** , *P*<0.01 compared with LMC.

Surprisingly, the total numbers of thymocytes are not significantly different at 6 weeks of age between transgenic mice and LMC (Table 1). At this time point, CD4^+^ thymocytes, double positive (DP) thymocytes and double negative (DN) thymocytes are almost identical in transgenic mice and LMC, while CD8 single-positive T cells are 2.3-fold higher in transgenic mice than LMC (p = 0.007, Table 1). CD44+ CD25+ double positive cell number is much higher in NOD.STAT5b mice than littermate control (9.4% verse 2.5%), suggesting STAT5 activity status (Fig. 2c). By 10 weeks of age, transgenic mice had slightly higher (1.6–2.0-fold) CD4^+^ T cells than LMC (Table 1). By 16 weeks of age, DN thymocytes dramatically increased in transgenic mice, resulting in a 5.7-fold increase compared to LMC (Table 1). In contrast, DP thymocytes in transgenic mice dramatically decreased by 12–16 weeks of age, resulting in a 30-fold reduction in transgenic mice (1.5 million or ∼2% of total thymocytes) compared to LMC (52.5 million or ∼70% of total thymocytes) (Table 1 and Fig. 2d). Interestingly, similar observations were made in mice that over-express a constitutively active Stat5b transgene and develop B cell lymphoma [27] but not in the B6 transgenic mice with the same non-activated form of Stat5b [28].

We also examined the cellular phenotypes in the spleen and thymus of NOD/B6 F1.Stat5b^Tg^ mice. Similar to the observations in NOD.Stat5b^Tg^ spleens, the number of CD4^+^ T cells is slightly increased in transgenic mice than LMC, while there were many more CD8^+^ T cells in the transgenic mice (54%) than LMC (7%) (Fig. 2e). Interestingly, the numbers and percentages of CD4^+^ and CD8^+^ thymocytes were similar in F1.Stat5b^Tg^ mice and LMC (Fig. 2e). These observations in the F1 mice are similar to the data reported for the Stat5b transgenic mice in the B6 background but different from the data for NOD.Stat5b^Tg^ mice. Our results, together with the previously published data [27], [28], suggest that Stat5b over-expression has differential effects on B, CD4 and CD8 T cell development. Furthermore, the impact of Stat5b transgene on CD8^+^ thymocytes is only observed in the NOD background, but not in the B6 or NOD/B6 F1 background, suggesting that the NOD genome can synergize with Stat5b to promote CD8^+^ thymocyte expansion.

### Rapid homeostatic proliferation of CD8^+^ T cells in NOD.Sta5b^Tg^ mice

To further characterize the phenotypes of the thymocytes from NOD.Stat5b^Tg^ mice, we examined a number of surface markers including CD1D, CD14, CD21, CD23, CD24, CD40, CD43, CD44, CD45, CD49b, CD51, CD54, CD69, CD70, CD80, CD83, CD86, CD95, CD95L, CD103, CD106, CD122, CD150, CD244.2, CCR5, PD-1, PD-L2 and CIRE. Most of these molecules did not exhibit significant differences between transgenic mice and LMC in the NOD genetic background (data not shown). The CD8^+^ thymocytes from 16 week old NOD.Stat5b^Tg^ mice showed profound up-regulation of CD44^+^CD122^+^ cells (Fig. 2f), two surface markers characteristic for both homeostatically proliferating naïve CD8^+^ T cells and CD8^+^ memory T cells. However, thymocytes from young transgenic mice or LMC (6 week old) comprised very few CD44^+^CD122^+^ cells (<1%). Interestingly, the splenic CD8^+^ T cell population already contained a relatively a high percentage of CD44^+^CD122^+^ T cells in young NOD.Stat5b^Tg^ mice (39%) and LMC (13%) (data not shown). At 16 weeks of age, the percentage of CD44^+^CD122^+^ CD8^+^ T cell population in the spleen remained similar to the observations in 6 week old mice (8% for LMC and 36% for transgenic mice, Fig. 2f). These results suggest that CD8^+^ T cells undergo rapid homeostatic expansion in both the thymus and peripheral lymphoid organs in NOD.Stat5b^Tg^ mice.

### STAT5 is increasingly phosphorylated over time in NOD.Stat5b^Tg^ mice

**Figure 3:**
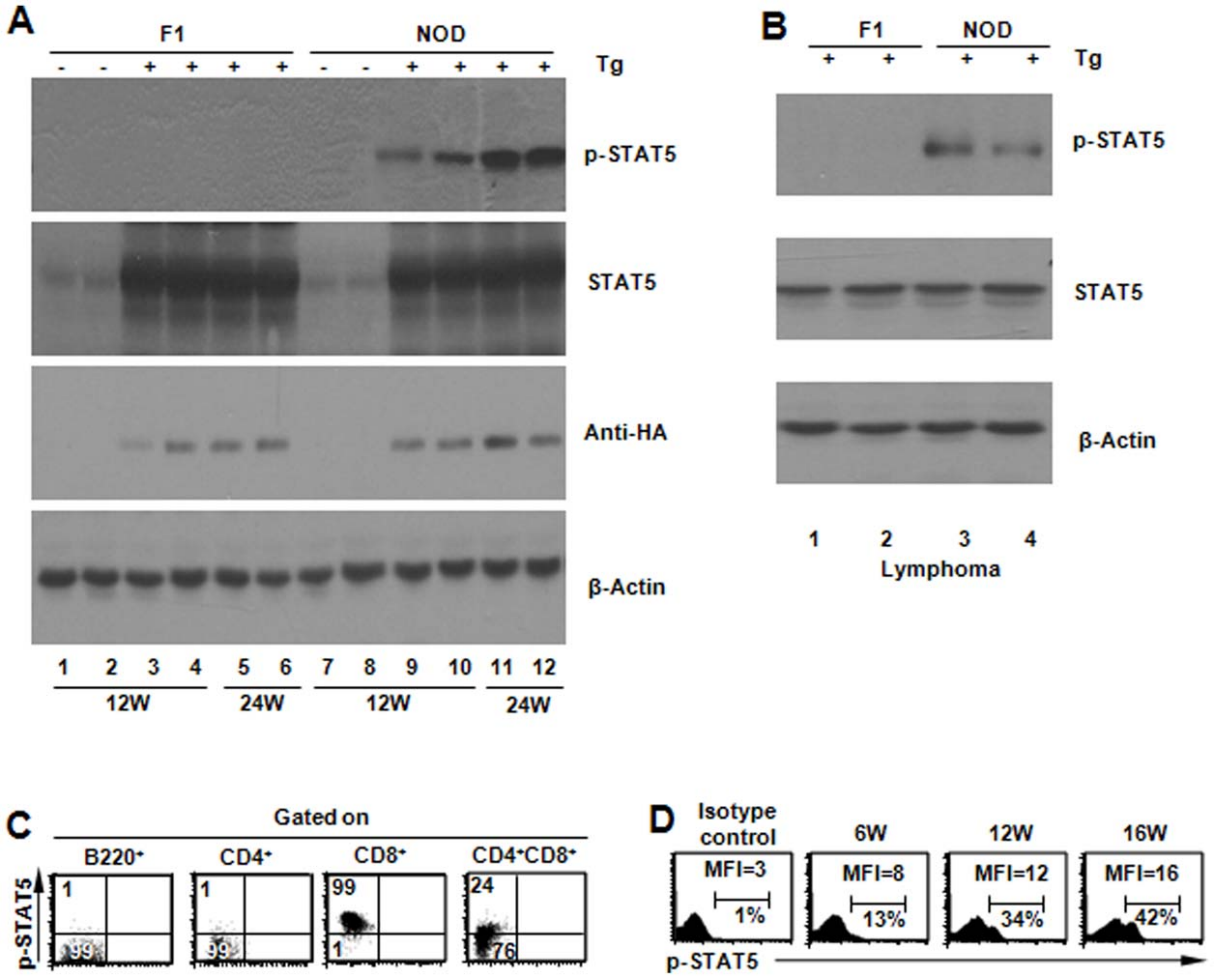
STAT5 phosphorylation in Stat5b transgenic mice. (A) Representative Western blotting analysis for phosphorylated and total STAT5 in F1 and NOD Stat5b transgenic lines. These mice did not have detectable signs of lymphoma based on physical examination and FACS analysis of T cell phenotypes. Protein extracts from thymus were used for the experiment. (B) Representative Western blotting analysis of phosphorylated STAT5 with thymus protein extracts from NOD.Stat5b transgenic mice with lymphoma. (C) STAT5 phosphorylation status in different cell types. Thymocytes and splenocytes from NOD Stat5b^Tg^ mice were analyzed by intracellular staining for phosphorylated STAT5 with an anti–pTyr694-STAT5 antibody. (D) FACS analysis showing progressive increase of STAT5 phosphorylation in thymocytes of NOD.Stat5b^Tg^ mice. Data for thymus are shown here for 6, 12 and 16 week old NOD.Stat5b^Tg^ mice (all without tumor). Representative data are shown from 1 of 3 similar experiments.

We next investigated why NOD.Stat5b^Tg^ mice developed T cell lymphoma in much higher incidence and at earlier ages compared to both B6 and NOD/B6 F1 mice even though the same Stat5b transgene is present in these three different transgenic strains. We hypothesized that differences in STAT5 phosphorylation may account for the observed differences in lymphoma incidences in these strains. We measured Stat5 phosphorylation (p-STAT5) using both FACS and Western blotting analysis. Similar to the observation in B6.Stat5b^Tg^ mice [1], STAT5 phosphorylation was not detected in F1.Stat5b^Tg^ mice without lymphoma (Fig. 3a) or with lymphoma (Fig. 3b), consistent with the observations of the low incidence of lymphoma observed in these strains. In contrast, Western blot and FACS analysis detected phosphorylated STAT5 protein at 6 weeks of age and increasing phosphorylation of STAT5 over time in the thymocytes of NOD.Stat5b^Tg^ mice (Fig. 3a& 3d). STAT5 phosphorylation was also detected in lymphoma cells from NOD.Stat5b^Tg^ mice (Fig. 3b).

In order to differentiate the cell types in which STAT5 is phosphorylated, we used FACS to analyze phosphorylated Stat5 in different cell types in the spleens and thymus of NOD.Stat5b^Tg^ mice. As shown in Fig. 3c, almost all CD8^+^CD4^−^ thymocytes stained positive for p-STAT5 and approximately one quarter of the CD8^+^CD4^+^ thymocytes also expressed, although at weaker levels, p- STAT5. However, CD4^+^CD8^−^ T cells and B cells had no detectable p-STAT5 (Fig. 3c), suggesting that phosphorylated Stat5, if present, is very low. FACS analysis also suggested that STAT5 phosphorylation in NOD.Stat5b^Tg^ mice gradually increased over time and was detectable at early ages, at least around six weeks of age. These results, together, suggest that STAT5 is selectively activated in CD8^+^ T cells in the NOD.Stat5b^Tg^ mice.

### Up-regulation of costimulatory molecules in NOD.Stat5b^Tg^ mice

**Figure 4:**
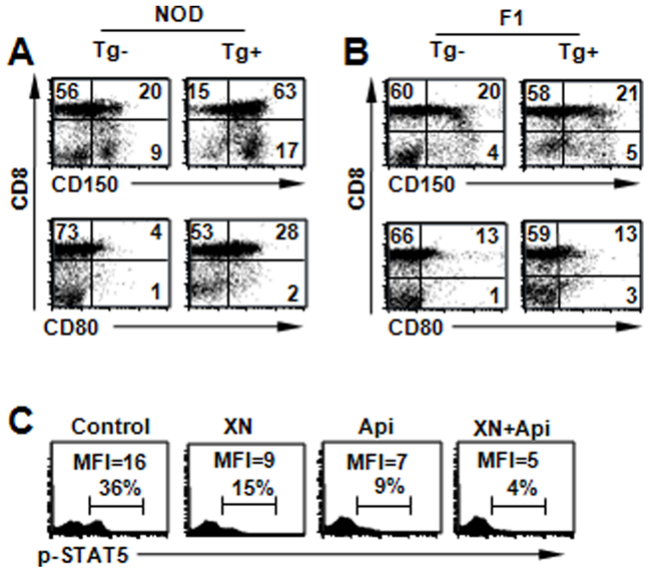
Expression of costimulatory molecules CD150, CD80 in Stat5b transgenic lines. (A) CD150 and CD80 are increased on the surface of CD8^+^ thymocytes even before the development of lymphoma. Shown are data from a NOD.Stat5^Tg^ mouse (Tg+) and a littermate control (Tg−) at 6 weeks of age. *P*<0.01, compared with that of littermate controls. (B) CD150 and CD80 are not significantly different between transgenic F1 mice and littermate controls at 6 weeks of age. *P*>0.05, compared with that of littermate controls. (C) p-STAT5 levels after two weeks of treatment with Apigenin and Xanthohumol alone or in combination. Data for thymus of the treated mice (12 weeks of age) are shown here. Representative data are shown from 1 of 3 similar experiments.

In addition to CD44 and CD122, two costimulatory molecules (CD80 and CD150) among the large number of surface molecules tested in this study were significantly up-regulated in CD4^+^CD8^+^ and CD4^−^CD8^+^ thymocytes of NOD.Stat5b^Tg^ mice but not in F1.Stat5b^Tg^ mice or their LMC (Fig. 4a & 4b). The expression patterns of the costimulatory molecules are consistent with the STAT5 phosphorylation pattern in these mice. CD150, also known as SLAM (Signaling Lymphocytic Activation Molecule), is a glycoprotein widely expressed at the transcriptional level in T, B, and myeloid cells and is rapidly up-regulated to the plasma membrane on activation of T cells and macrophages [29]. CD80 is expressed in multiple cell types, including B cells, T cells, macrophages, and dendritic cells (DC) which provides a costimulatory signal necessary for T cell activation and survival [30].

### NOD.Stat5b^Tg^-specific transcriptional alterations

**Figure 5:**
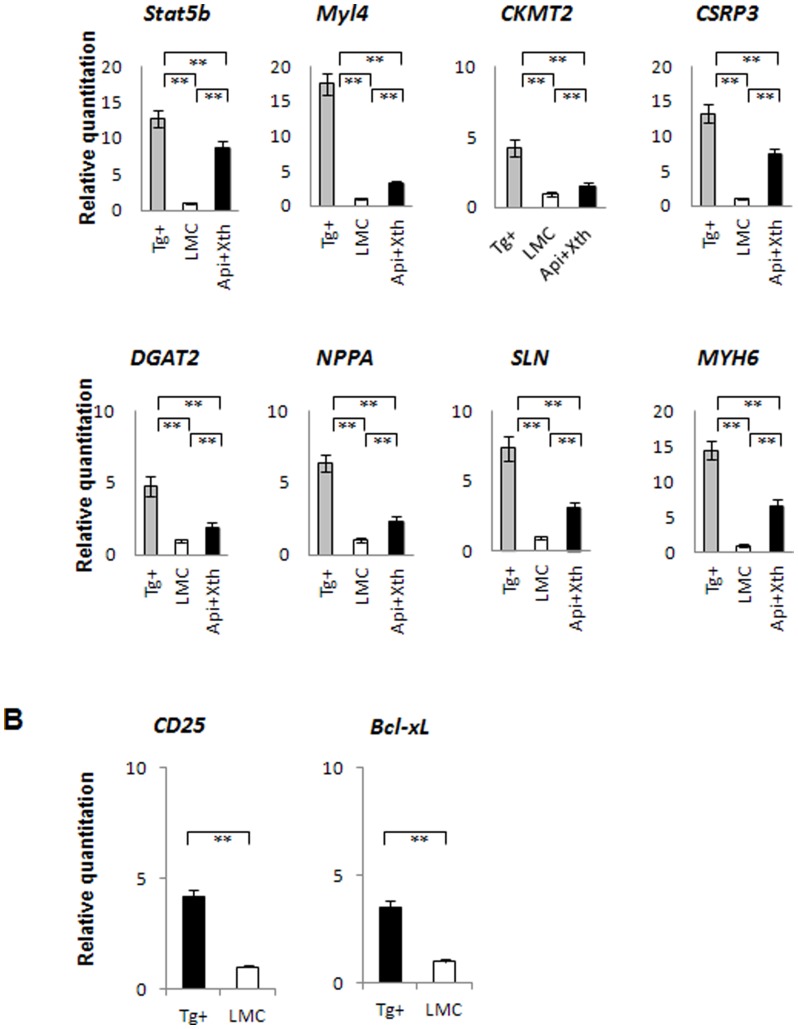
Up-regulation of gene expression in NOD.Stat5b^Tg^ mice. (A)Real-time RT-PCR was used to analyze RNA from thymus of NOD.Stat5b^Tg^ (Tg+) mice and their littermate controls (LMC) (4 weeks of age) as well as NOD.Stat5b^Tg^ mice after treatment with both Apigenin and Xanthohumol (Api+XN). RNA concentration was normalized with GAPDH. (B) Real-time RT-PCR was used to analyze RNA from thymus of NOD.Stat5b^Tg^ (Tg+) mice and their littermate controls (LMC) (8 weeks of age). Data from LMC were arbitrarily set to “1” for easier comparison. **, *P*<0.01, compared with that of littermate controls.

To further dissect the molecular mechanisms underlying Stat5b -mediated lymphomagenesis, we conducted a global gene expression analysis using the Illumina gene chips with thymus tissues from four groups of mice at 4 weeks of age: 1) NOD.Stat5b^Tg^, 2) non-transgenic littermates of NOD.Stat5b^Tg^, 3) F1 (B6xNOD).Stat5b^Tg^, and 4) non-transgenic littermates of the F1 transgenic mice. None of the mice had detectable tumor as evaluated by physical examination or FACS analysis. The microarray data have been deposited in NCBI Gene Expression Omnibus and are accessible through GEO Series accession number GSE31526. Since NOD.Stat5b^Tg^ mice develop lymphoma at high incidence and the F1.Stat5b^Tg^ mice have very low incidence, we hypothesized that the genes altered in NOD.Stat5b^Tg^ mice but not in the F1.Stat5b^Tg^ mice might be excellent candidate genes implicated in lymphomagenesis. The number of differentially expressed genes between transgenic and non-transgenic mice was relatively small, with 37 genes that fulfill our selection criteria (Table 2). These genes are implicated in cell proliferation, migration, energy production and many of these genes are also altered in various cancer patients. Real-time RT-PCR was used to confirm the expression differences for representative genes suggested by microarray (Fig. 5a) as well as two genes known to be regulated by Stat5 (CD25 and Bcl-xL) (Fig. 5b).

**Table 2:** Genes up-regulated in the NOD.Stat5b^Tg^ mice but not in the F1.Stat5b^Tg^ mice.

Genes	NOD Tg/LMC*	F1 Tg/LMC*	Function
*MYL4*	9.2	0.7	Migration
*MYH6*	6.3	0.7	Migration
*ACTC1*	3.5	0.7	Migration
*TNNC1*	4.0	0.8	Migration
*Mybphl*	4.0	0.9	Migration
*MYL7*	3.3	0.9	Migration
*MYL1*	2.7	0.4	Migration
*NPPA*	5.8	0.9	Migration
*GLYCAM1*	3.5	1.6	Migration
*SLN*	6.6	0.7	Energy
*ATP2A2*	2.7	0.8	Energy ↑
*COX8B*	2.8	0.4	Energy ↑
*COX7A1*	2.7	0.6	Energy
*COX6A2*	3.3	1.3	Energy
*MB*	4.9	0.8	Energy
***DGAT2***	2.7	0.8	Energy
*GPD1*	2.5	0.8	Energy ↑
*UCP1*	3.6	0.5	Lipid ↑
*SCD1*	3.0	0.7	Lipid
*ACOT11*	2.2	1.0	Lipid
*CSRP3*	4.4	0.8	Proliferation
***CKMT2***	3.1	0.8	Proliferation ↑
*THRSP*	3.5	0.8	↑ in cancer
*CIDEA*	2.5	0.6	↑ in cancer
*ANKRD1*	2.9	0.9	↑ in cancer
*DDC*	4.6	1.9	↑ in cancer
*TCAP*	2.5	1.0	↑ in cancer
*RETNLA*	3.9	1.7	↑ in cancer
*CD163l1*	6.4	1.8	↑ in cancer
*MOD1*	2.0	0.8	
*BC018222*	3.0	0.8	
*LEP*	2.3	0.8	
*CDO1*	2.2	0.8	↑ in cancer
*ACTC1*	2.3	0.9	↑ in cancer
*PPP1R3C*	2.1	0.9	↑ in cancer
*AGPAT2*	2.1	1.0	
*FBLN1*	2.0	1.1	↑ in cancer

* Fold change. Tg = transgenic, LMC = littermate control. ↑ increased in cancer patients. All genes have a fold change greater than 2 and false positive rate less than 1%.

### Lymphomagenesis correlates with Stat5 phosphorylation and Stat5b pathway gene expression

Apigenin and Xanthohumol are two small molecules contained in plants and have been reported to possess chemopreventive properties through multiple pathway [31], [32]. They have potential inhibitory effects on JAK/STAT signaling pathway [33], [34] and DGAT2 activation [32]. Therefore, they were tested for their potential ability to prevent lymphoma in the NOD.Stat5b^Tg^ mice. Treatment started at 5 weeks of age with three cycles of 3-week on and 3-week off. The treated mice were free of lymphomas by 30 weeks of age (Fig. 1).

To investigate the molecular mechanism underlying chemoprevention of lymphomagenesis, we carried out a new experiment to treat 12 weeks old NOD.Stat5b^Tg^ mice with Apigenin and Xanthohumol, separately or in combination. After two weeks of treatment, FACS analysis of thymocytes revealed reduced p- STAT5 in the treated mice (Fig. 4c). Furthermore, the expression of genes up-regulated in the NOD.Stat5b^Tg^ mice (Creatine kinase, mitochondrial 2 (*Ckmt2*), diacylglycerol O-acyltransferase 2 (*Dgat2*), Myosin, light chain 4 (*Myl4*), Cysteine and glycine-rich protein 3 (*Csrp3*), natriuretic peptide A (*Nppa*), sarcolipin (*Sln*) and myosin, heavy chain 6 (*Myh6*)) were also significantly decreased by the chemoprevention agents (*p*<0.01) but not completely normalized to the levels observed in LMC (Fig. 5a). These results suggest that reduction of these genes correlates with the prevention of lymphomagenesis in NOD genetic background.

## Discussion

Demonstration of a critical role of Stat5b in hematologic malignancies has generated increasing interest in the mechanisms through which Stat5b functions as an oncogene [35]. Previous studies using a constitutively activated Stat5b transgenic mouse model (Stat5b-CA) have suggested important roles for STAT5 in T- and B-cell development. In this model, persistent STAT5 activation was shown to be weakly oncogenic, leading to the late emergence of clonal B-cell lymphoma/leukemia at a low incidence [27]. Furthermore, activated STAT5 was found to cooperate with the loss of function of the p53 tumor suppressor gene to both accelerate disease onset and to skew the large tumor spectrum that normally characterize p53-deficient mice to strongly favor B-cell lymphoma/leukemia [36]. These results suggested that STAT5 activation is critical to its oncogenic properties. However, this idea was contrasted by the observations in a different Stat5b transgenic B6 mouse line which expresses a wild type Stat5b. Even though Stat5b phosphorylation was not detected in the B6.Stat5b^Tg^ mice, CD8^+^ T cell lymphoma was observed in about 12% of the transgenic mice [1]. The molecular mechanism underlying lymphomagenesis in this model remained elusive. Recently, it was reported that the CD8 T cell number is particularly sensitive to gene dosage of STAT5 [37].

We have previously shown that the NOD mice harbor a mutant Stat5b with weaker DNA binding affinity [38], [39]. It was later found that constitutive Stat5b phosphorylation was higher in NOD mice [22]. We have recently reported that NOD Stat5b transgenic mice have significantly lower incidence of type 1 diabetes than NOD mice and the levels of CD4^+^ regulatory T cells are increased in the Stat5b transgenic NOD mice [23]. Since Stat5 plays a critical role in CD4^+^ regulatory T cell development, we hypothesized that transgenic Stat5b in the NOD genetic background may exacerbate lymphomagenesis. Our prediction was proven correct by the dramatically increased incidence and acceleration of lymphoma in NOD.Stat5b^Tg^ mice. Comparative studies on the molecular and cellular phenotypes for the NOD.Stat5b^Tg^ mice, which have a high lymphoma incidence, and the B6.Stat5b^Tg^ and NOD/B6 F1.Stat5b^Tg^ mice which have low incidence of lymphoma, shed new lights on the role of Stat5b and the NOD genetic background on lymphomagenesis. In all three models, Stat5b can modestly induce CD8^+^ T cell homeostatic proliferation in the spleen and has weak effect on B cell and CD4^+^ T cell proliferation in the spleen and thymus. However, the Stat5b transgene profoundly promote homeostatic expansion of CD8^+^ thymocytes only in the NOD.Stat5b^Tg^ mice in which Stat5b is strongly phosphorylated. These results suggest that phosphorylation, or at least strong phosphorylation, may not be necessary for Stat5b to promote lymphocyte proliferation while the progressively increasing phosphorylation of STAT5 observed in NOD.Stat5b^Tg^ mice but not in F1 or B6 Stat5b transgenic mice may account for the differences in lymphoma incidences observed in these models. The precise reasons why STAT5 is strongly phosphorylated in the NOD genetic background but not phosphorylated, or only weakly phosphorylated in the B6 genetic background are still unknown. Certainly, this must be related to the genetic variations in NOD genome. Indeed, it has been reported that a diabetes associated genetic interval on chromosome 11 and other genetic intervals may be associated with the increased baseline Stat5b phosphorylation in NOD mice [21], [22]. Interestingly, significantly increased IL-2Rβ on CD8^+^ T cells and serum IL-2 concentration were observed in NOD.Stat5b^Tg^ mice (data not shown). IL-2Rβ is regulated by Stat5b and reciprocally IL-2/IL-2Rβ can induce STAT5 phosphorylation. Meanwhile, other Stat5b-activating cytokines and growth factors may also be implicated in inducing STAT5 phosphorylation in NOD mice.

Stat5b has a much more profound influence on CD8^+^ T cell proliferation than CD4^+^ T cells and B cells in both B6 and NOD genetic background. The key difference between the strains is the phosphorylation of Stat5 that occurs selectively in CD8^+^ T cells in our NOD.Stat5b^Tg^ mouse model. These results suggest that Stat5 phosphorylation is critical to lymphomagenesis. The second key difference between the strains is the rapid homeostatic proliferation of CD8^+^ thymocytes in the NOD but not in the B6 or F1 background, suggesting that other genes in the NOD genome synergizes with the transgenic Stat5b to induce CD8^+^ thymocytes proliferation. These results are consistent with the hypothesis on the thymus origin of T cell lymphoma [17], [18].

The most likely mechanisms through which Stat5b could exert effects on expansion of T-lineage cells is through cooperation with pre-TCR [40], [41], [42], TCR [43], [44] and costimulatory signals or through its actions in transmitting signals from cytokines [45]. We observed significantly higher expression of several costimulatory molecules such as CD150 and CD80 over time in thymocytes of NOD.Stat5b^Tg^ mice but not in B6 or F1.Stat5b^Tg^ mice. CD80 and CD150 do not appear to be direct target genes for Stat5b as Stat5 consensus sequence motifs are not identified in their promoters.

The Stat5b transgene in the NOD genetic background also alters the expression levels of a number of genes relevant to tumorigenesis as suggested by our global transcription analysis that focused on the early molecular events responsible for the initiation of lymphomagenesis. Many of these genes are not only influenced by the presence of the Stat5b transgene but also the NOD genetic background as they are only altered in the NOD.Stat5b^Tg^ mice but not in the F1.Stat5b^Tg^ mice or the non-transgenic littermates of both strains. The potential role of these genes and proteins are further substantiated by the chemoprevention experiments using apigenin and Xanthohumol, which significantly down-regulated the expression of STAT5 phosphorylation and Stat5 pathway genes. This experiment also further demonstrated importance of Stat5 activation for the initiation of CD8^+^ T-cell malignancy

In summary, this study demonstrated that genetic background is critical to the Stat5b-mediated lymphomagenesis through regulation of selective Stat5b activation in CD8+ thymocytes, which results in the alteration of many tumorigenic genes. Chemopreventive compounds that are able to reduce Stat5 phosphorylation can efficiently block lymphomagenesis, demonstrating that the NOD.Stat5b^Tg^ mouse is an excellent model for testing novel chemopreventive strategies.

## Materials and Methods

### Mice and lymphoma prevention experiments

The *Stat5b* transgenic (Tg) mouse in the B6 genetic background (B6.Stat5b^Tg^) was generated as previously described by Kelly et al [1]. The Stat5b transgene was under the control of the H-2K^b^ promoter and heavy-chain enhancer with HA-tag as previously described [28]. The transgene was moved from the B6 to the NOD genetic background through 21 generations of backcrossing in our laboratory. The resulting strain (NOD.Stat5b^Tg^) was used for experiments described in this study. NOD/B6 F1 mice used in this study were obtained by breeding the NOD.Stat5b^Tg^ mice with regular B6 mice from the Jackson Laboratory.

In several experiments, mice were monitored for tumors through physical examination. Mice showing sufficient signs of pain and suffering including thymic and cervical lymphoid enlargement were euthanized for a complete necropsy. Final diagnosis of disease was established by clinical, pathologic and FACS analysis. Images of H&E staining were visualized with an Axiophot I microscope (Carl Zeiss). Images were captured with a Nikon digital sight DS-U1 camera, and treated on a computer with NIS-Elements F3.0 software. Xanthohumol and apigenin (>98% purity by HPLC) purchased from Sigma (St. Louis, MO, USA) were used in the chemoprevention experiments. Xanthohumol (100 mg/kg/day) and Apigenin (200 mg/kg/day) were injected intraperitoneally into 16 NOD.Stat5b^Tg^ mice as a single dose. Treatment started at 5 weeks of age with three cycles of 3-week on and 3-week off. The treated mice were monitored for 30 weeks.

All experiments were performed under an Animal Component of Research Protocol (ACORP) approved by the Institutional Animals Care and Use Committees (IACUCs) of the Georgia Health Science University.

### Flow cytometry

Single-cell suspensions from thymus, spleen and lymph nodes were stained and analyzed using a fluorescence-activated cell sorting (FACS) flow cytometer (BD Biosciences) with CELLQuest 3.3 software (Becton Dickinson, San Jose, CA). The following antibodies, all from BD PharMingen (San Diego, CA), were used: anti-CD4–FITC, anti-CD4–PE, and anti-CD4– Percp; anti-CD8–FITC, anti-CD8–PE, and anti-CD8–APC; anti–CD45R-FITC; anti–CD19-PE; anti-CD25–APC and anti–IL-2R beta–GFP; anti-CD44–PE; anti-CD3–GFP; anti-CD1D-PE; anti-CD14-PE, anti-CD21 PE; anti-CD23-PE; anti-CD24-PE; anti-CD40 PE; anti-CD43-PE; anti-CD44-PE; anti-CD45-PE; anti-CD49b-PE; anti-CD51-PE; anti-CD54-PE; anti-CD69-PE; anti-CD70-PE; anti-CD80-PE; anti-CD83-PE; anti-CD86-PE; anti-CD95-PE; anti-CD95L-PE; anti-CD103-PE; anti-CD106 PE; anti-CD122-PE; anti-CD150-PE; anti-CD244.2-PE; anti-CCR5-PE; anti-PD-1-PE; anti-PD-L2-PE and anti-CIRE-PE. For intracellular staining, cells were fixed and permeablized using Cytofix/Cytoperm solution (BD PharMingen), followed by P-Stat5(pY694)-APC staining.

### RNA Purification and gene expression studies

RNA was isolated from thymus using the miRACLE™ total RNA isolation kit (Jinfiniti Biosciences) or RNeasy® kit (QIAGEN) and used for microarray and real-time RT-PCR analyses. The Illumina's MouseRef-8 v2.0 Expression BeadChip was used for transcriptional analysis. This BeadChip targets approximately 25,600 well-annotated RefSeq transcripts, over 19,100 unique genes, and enables the interrogation of eight samples in parallel. Probe preparation and hybridization were carried out according to Manufacture's recommendation. The microarray data are MIAME compliant and have been deposited in NCBI Gene Expression Omnibus and are accessible through GEO Series accession number GSE31526.

Differential expression analyses were conducted using the LIMMA (Linear Models for Microarray Analysis) package from the Bioconductor project [46]. LIMMA uses an empirical Bayes approach that uses the variability in all genes for testing for significant differences, with this approach resulting in more stable inferences for a relatively small number of arrays [46], [47]. We used the false discovery rate (FDR) adjustment for multiple testing [48] and a FDR cutoff of 1% is considered significant.

Real-time PCR was carried out in 96-well PCR plates using the ABI Prism 7900HT real-time PCR instrument (Applied Biosystems, Roche). The following primers, all from Applied Biosystems (Carlsbad, CA), were used: *Stat5b*, *Ckmt2*, *Dgat2*, *Myl4*, *Csrp3*, *Nppa*, *Sln* and *Myh6.* A final volume of 20 µl was used containing 2 µl of cDNA, 10 µl of TaqMan Master Mix (Roche, Minneapolis), 2 µl mix containing primers (200 nM of each primer, except for reference primers [20 nM]), 2 µl mix containing probes (100 nM of each probe), and water. The reaction was subjected to denaturation at 95°C for 2 min, followed by 40 cycles of denaturation at 95°C for 45 s and annealing/elongation at 60°C for 1 min. Samples were tested in triplicate and negative and positive controls were included with each run. The fluorescent signal was measured at the end of the annealing/elongation step in each cycle.

### Western blotting

Whole cell extracts were prepared from thymus or spleens using M-PER mammalian protein extraction reagent supplemented with Halt protease and phosphatase inhibitor cocktail (Thermo scientific). Western blot analysis was carried out as reported [49] by probing the blots with rabbit anti-phospho-Stat5 (Tyr694) (SC-101806), rabbit anti-Stat5 (SC-835), mouse anti-HA (SC-7392) or mouse anti-β-actin (SC-69879) (Santa Cruz biotechnology), washed, and incubated with a horseradish peroxidase-conjugated anti–rabbit or anti–mouse antibody (Santa Cruz biotechnology). Immunopositive bands were photographed (Versadoc; Bio-Rad) after Blots were developed with an enhanced chemiluminescent substrate (Pierce Chemical Co).

### Statistics

Pair-wise comparisons were made with Student t test. Three way comparisons are carried out using ANOVA with post-hoc tests. A probability (*p*) value was set at 0.05 for statistical significance. The Log Rank test was used to assess statistical significance for the tumor incidence difference.
